# Adjunctive Semaglutide in Patients Undergoing Intragastric Balloon for Weight Loss: 12-Month Prospective Comparative Study

**DOI:** 10.1007/s11695-025-08368-5

**Published:** 2025-11-10

**Authors:** Khaled E. Barakat, Doaa K. Abuhasan, Mohamed F. Asal, Ahmed Adham R. Elsayed, Mohamed R. Mahmoud, Madeline Guy, Rama Safadi, Marc D. Basson

**Affiliations:** 1 Surgery, Alexandria University Faculty of Medicine, Alexandria, Egypt; 2 Surgery, Borg Al Arab New Hospital, Alexandria, Egypt; 3 Medical Education, Alexandria University Faculty of Medicine, Alexandria, Egypt; 4 Biomedical Sciences, Northeast Ohio Medical University, Rootstown, USA; 5 Surgery, Northeast Ohio Medical University, Rootstown, USA; 6 College of Medicine, Northeast Ohio Medical University, Rootstown, USA

**Keywords:** Obesity, Semaglutide, GLP-1 agonist, Intragastric balloon, Weight loss, Weight regain

## Abstract

**Introduction:**

Given the global increase in obesity prevalence, there has been an emergence of a multitude of treatment options, specifically less invasive operations, like intragastric balloons, and pharmaceutical treatments like semaglutide. The aim of this study is to evaluate the synergistic effects of the intragastric balloon and semaglutide on weight reduction and weight regain.

**Methods:**

In this prospective, randomized cohort, adults between the ages of 18 and 65, with a BMI of at least 27 kg/m², were assigned to one of two treatment groups: IGB only or IGB + semaglutide. Subcutaneous injections of semaglutide were administered with increasing dosages on a weekly basis in the second month and were continued after the removal of the intragastric balloon. All participants were monitored, and results were recorded at 3, 6, and 12 months.

**Results:**

Forty patients completed the study (*n* = 20 per group). The IGB + semaglutide group lost more weight than the IGB only group at 3, 6, and 12 months, with statistically significant differences at 6 months (29.09 ± 3.45 kg vs. 18.35 ± 2.80 kg, *p* < 0.001) and 12 months (33.03 ± 3.55 kg vs. 15.56 ± 2.50 kg, *p* < 0.001). After intragastric balloon removal at 6 months, the IGB only group regained previously lost weight while the IGB + semaglutide group continued to lose weight (2.79 ± 1.74 vs. -3.94 ± 2.16, *p* < 0.001).

**Conclusion:**

Adjunctive semaglutide therapy with intragastric balloon (IGB) optimizes weight loss, while enhancing the sustainability achieved with intragastric ballooning alone. This combined therapeutic approach may provide an additional non-invasive intervention that provides optimal results and long-term weight loss maintenance.

**Graphical Abstract:**

Created in BioRender. Basson, M. (2025) https://BioRender.com/y0a16l4

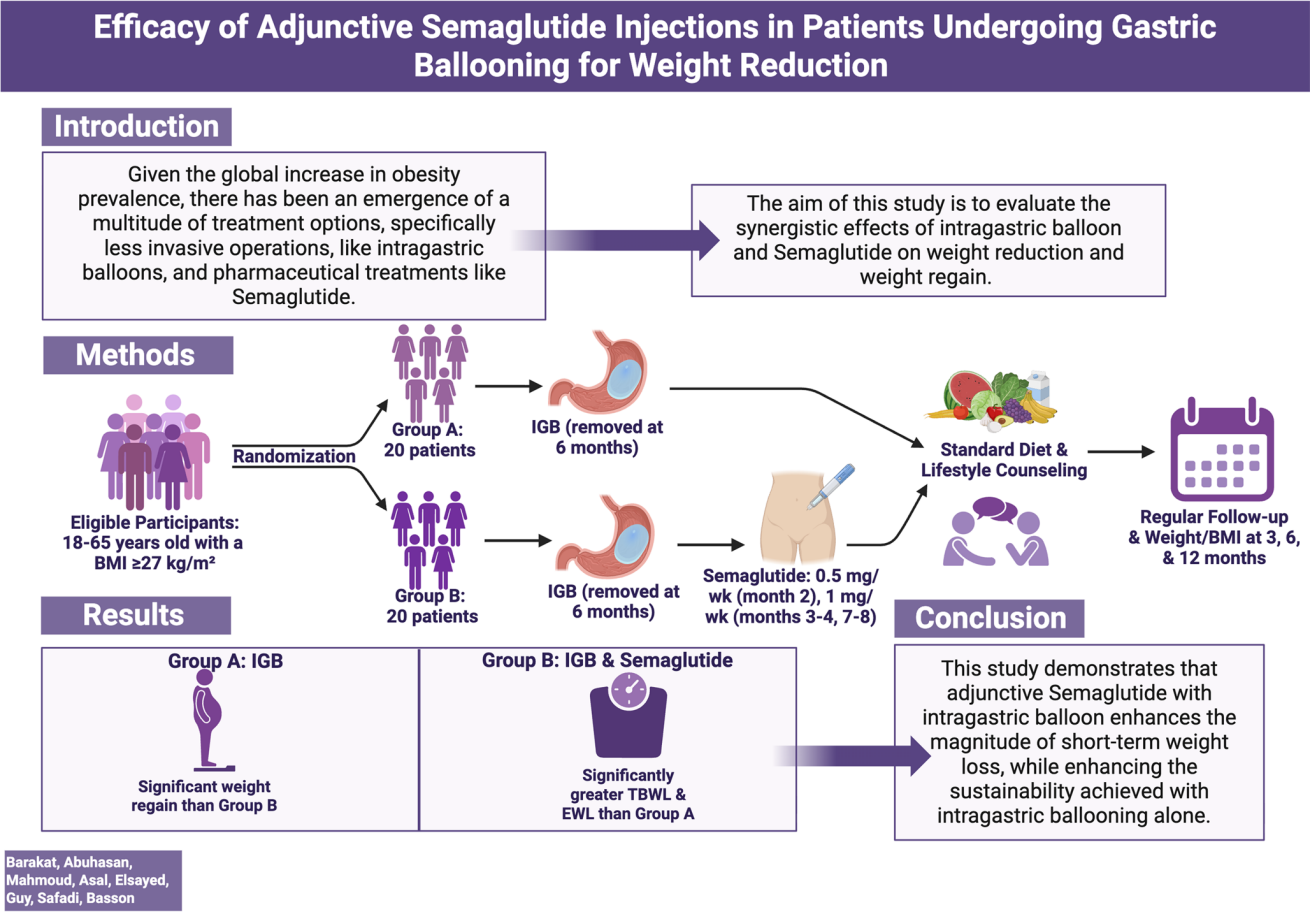

## Introduction

Obesity represents a global public health burden. It is typically interrelated to a plethora of complications that predispose to avoidable morbidity and mortality [1]. The rates of obesity worldwide have more than doubled over the past 35 years, with adolescent obesity having quadrupled [2]. While lifestyle intervention remains a cornerstone in the management of obesity, its effectiveness is often modest, with long-term studies such as Look AHEAD showing that approximately 50% of participants achieved ≥ 5% weight loss and almost one-third of participants achieved ≥ 10% weight loss at 8 years. These outcomes depend heavily on sustained patient engagement and adherence to behavioral changes [3]. Obesity remains an underdiagnosed and undertreated condition [4]. Classically, treatment is initiated from the least invasive and may be escalated towards pharmacotherapy, endoscopic bariatric interventions, and lastly, surgical intervention.

At present, the gold standard management for the unsuccessful medical treatment of obesity is surgical intervention, providing unrivalled weight loss. These interventions include but are not limited to the adjustable gastric band, sleeve gastrectomy, and Roux-en-Y gastric bypass [5]. Despite providing considerable weight loss, only between 0.5 and 1% of eligible patients undergo bariatric surgery [6]. Perhaps this may be in part due to high cost, concerns related to surgical intervention, and lack of access to bariatric centers [7]. Endoscopic bariatric therapy offers a less invasive option for patients reluctant to undergo surgery and includes various procedures such as space-occupying devices, restrictive procedures, and aspiration therapies [8]. Space-occupying devices refer to Intragastric balloons (IGB) that are usually temporary, endoscopically placed air or fluid-filled balloons, resulting in fullness that leads to weight loss [8]. 

Semaglutide, a glucagon-like peptide-1 receptor agonist (GLP-1 RA), has emerged as an effective medical treatment alternative for obesity. Its subcutaneous administration has demonstrated significant success in promoting weight loss in adults with type 2 diabetes [9]. It is approved as a supplemental therapy for overweight and obese adults, in conjunction with a calorie-restricted diet and enhanced physical activity in adults with a baseline BMI ≥ 30 or ≥ 27 kg/m² with at least one weight-related comorbidity, across the USA, Canada, Europe, and the UK [10]. 

This study explores the use of intragastric balloons (IGBs), highlighting their applications, efficacy, and role in the management of obesity. While IGBs are an established endoscopic intervention for weight loss, the impact of combining IGB insertion with semaglutide, a GLP-1 receptor agonist, remains unclear. Therefore, we aim to evaluate the efficacy of this combined approach on weight reduction.

## Methods

### Study Design and Setting

This prospective comparative study was conducted at Alexandria Main University Hospital, Alexandria, and included adult patients diagnosed with overweight or obesity who were scheduled for endoscopic intragastric balloon (IGB) placement.

### Patient Selection

Eligible participants were men and women aged 18 to 65 years with a body mass index (BMI) of 27 kg/m² or higher. All patients provided informed consent prior to enrollment. Exclusion criteria comprised a history of previous bariatric surgery, contraindications to IGB insertion or semaglutide use, the presence of gastrointestinal disorders such as inflammatory bowel disease or gastroparesis, severe organ dysfunction (renal, hepatic, or cardiac), pregnancy, and current use of other anti-obesity medications.

### Randomization and Group Allocation

Following screening and informed consent, patients were randomized into two equal groups, with 25 participants allocated to each group. Study recruitment was closed once complete 12-month follow-up data were obtained for 20 patients in each group. The random allocation sequence was generated through an online tool (http://www.randomizer.org), which generated a randomized list of patient identification codes. Allocation was concealed using sealed, opaque envelopes, prepared and managed by an independent third party to prevent selection bias. IGB only, the control group, underwent intragastric balloon (IGB) insertion alone, while the intervention group received IGB insertion in combination with subcutaneous semaglutide injections. This was an open-label trial; hence, neither participants nor investigators were blinded to group assignments. The nature of the interventions precluded effective blinding.

## Intervention Protocol

All IGB procedures were performed under conscious sedation by an experienced endoscopist, using endoscopically placed, liquid-filled intragastric balloons (Spatz3 ^®^) with a fill volume ranging from 400 to 700 ml of saline and methylene blue, adjusted according to the fundus size as assessed by endoscopy. The IGB was maintained intragastric for six months, after which it was removed endoscopically.

Patients in the IGB + semaglutide group received the Semaglutide (Ozempic ^®^) injections following a structured titration and administration schedule. The regimen began with an initial dose of 0.5 mg once weekly during the second month. The dose was then escalated to the full therapeutic dose of 1 mg once weekly during the third and fourth months. This dose was maintained during this period, then paused for the fifth and sixth months while the intragastric balloon was still in place and subsequently removed after the sixth month. Semaglutide was reintroduced at 1 mg once weekly during the seventh and eighth months, after which the therapy was discontinued. All participants in both groups were instructed to adhere to a standardized dietary protocol, beginning with a liquid diet in the first week post-IGB insertion, progressing to a soft diet, and subsequently transitioning to a structured hypocaloric solid diet. Dietary counseling and lifestyle modification support, including recommendations for regular physical activity, were provided by a dietitian throughout the study period.

### Semaglutide Regimen

The Semaglutide dosing regimen in this study was tailored to optimize the synergistic effects with the intragastric balloon while maintaining patient safety and tolerability. Starting with an initial dose of 0.5 mg weekly allowed gradual dose escalation to the therapeutic dose of 1 mg weekly, minimizing the risk of adverse effects commonly associated with higher doses. The decision to avoid reaching the maximum dose of 2 mg was based on the hypothesis that 1 mg would provide sufficient efficacy when combined with the gastric balloon. The dosing schedule was strategically designed to be given in the critical period of balloon placement, specifically during the second to fourth months and after balloon removal in the seventh and eighth months. While the maximum effect of the balloon is observed in the first month, our intention was to support and sustain the balloon’s weight loss effect after this initial peak period (2nd to 4th months). Continued Semaglutide adaptations and on for 2 months post-balloon removal aimed to sustain weight loss, support metabolic adaptations, and facilitate behavioral changes necessary for long-term maintenance (7th & 8th months). The dosing schedule includes a 2-month hiatus during the fifth and sixth months, corresponding with the continuation of gastric balloon therapy during a total 6-month period. This pause was intentional and designed to support and sustain balloon efficacy after the initial phase, allowing the Semaglutide to be reintroduced after balloon removal in the seventh and eighth months to consolidate weight loss and support behavioral change. This regimen balances efficacy, safety, and patient adherence by providing targeted pharmacological support aligned with the clinical course of gastric balloon therapy.

#### Outcome Measures and Follow-Up

- Primary outcome:

The percentage change in body weight from baseline to 12 months after intervention is the primary outcome investigated in this study. Weight and BMI were measured at baseline and then at 3, 6, and 12 months. Total Body Weight Loss (TBWL) and Excess Weight Loss (EWL) are the two principal metrics used to assess the effectiveness of weight loss, especially in a bariatric setting. TBWL is the absolute reduction in body weight, calculated as a percentage of the preoperative weight by dividing the difference between the initial and current weights by the initial weight, and then multiplying by 100. In contrast, EWL is the proportion of weight loss relative to the excess weight above an “ideal weight,” using the formula: (preoperative weight minus current weight) divided by (preoperative weight minus ideal weight), also multiplied by 100. The “ideal weight” refers to the body weight corresponding to a body mass index (BMI) of 25 kg/m², calculated as 25 times the square of the person’s height in meters.

- Secondary Outcome:

The secondary outcomes investigated in the present study included safety, specifically the incidence and nature of treatment-related complications associated with the intragastric balloon and Semaglutide combination therapy. This also includes monitoring adverse events such as nausea, vomiting, gastroparesis, pancreatitis, and any other clinically relevant complications throughout the study duration.

- Follow up:

Adherence to the treatment protocol, patient-reported tolerance, serious complications, and any adverse events were prospectively recorded at each scheduled visit, although a specific validated nausea scoring tool was not employed. Clinical assessments and patient self-reports guided the monitoring process. We define serious complications as any adverse events requiring discontinuation of the current plan, hospitalization, or posing significant health risks occurring within the study follow-up period of 12 months. The primary endpoint was defined as the difference in mean weight loss between the two groups at the end of the twelve-month follow-up period.

### Sample Size Calculation

The sample size was calculated based on detecting a clinically meaningful difference in mean weight loss between the IGB-only group and the IGB + Semaglutide at the 6-month follow-up. Assuming a mean difference of approximately 10.74 kg (29.09 kg vs. 18.35 kg), with a standard deviation of approximately 3.5 kg based on preliminary data and prior literature, a two-sided alpha level of 0.05 and 80% power, the minimum required sample size per group was estimated to be 17 participants. To account for potential dropouts and losses to follow-up, 25 participants were initially recruited per group, ensuring that at least 20 completed the study in each arm, thus maintaining sufficient power for the final analysis. This sample size is justified to detect the significant differences observed in weight loss outcomes and supports the validity of the study findings.

### Statistical Analysis of the Data

Data were analyzed using the IBM SPSS software package version 20.0. (Armonk, NY: IBM Corp, released in 2011). Categorical data were represented as numbers and percentages. The chi-square test was applied to compare the two groups. Alternatively, Fisher’s Exact correction test was applied when more than 20% of the cells had an expected count less than 5. For continuous data, they were tested for normality by the Shapiro-Wilk test. Quantitative data were expressed as range (minimum and maximum), mean, standard deviation, and median. Student t-test was used to compare two groups for normally distributed quantitative variables, while ANOVA with repeated measures was used for normally distributed quantitative variables, to compare between more than two periods or stages, and Post Hoc test (Bonferroni adjusted) for pairwise comparisons. On the other hand, the Mann-Whitney test was used to compare two groups for not normally distributed quantitative variables. The significance of the results obtained was judged at the 5% level. An intention-to-treat (ITT) analysis was performed, including all randomized participants regardless of dropout status.

## Results

**Fig. 1:**
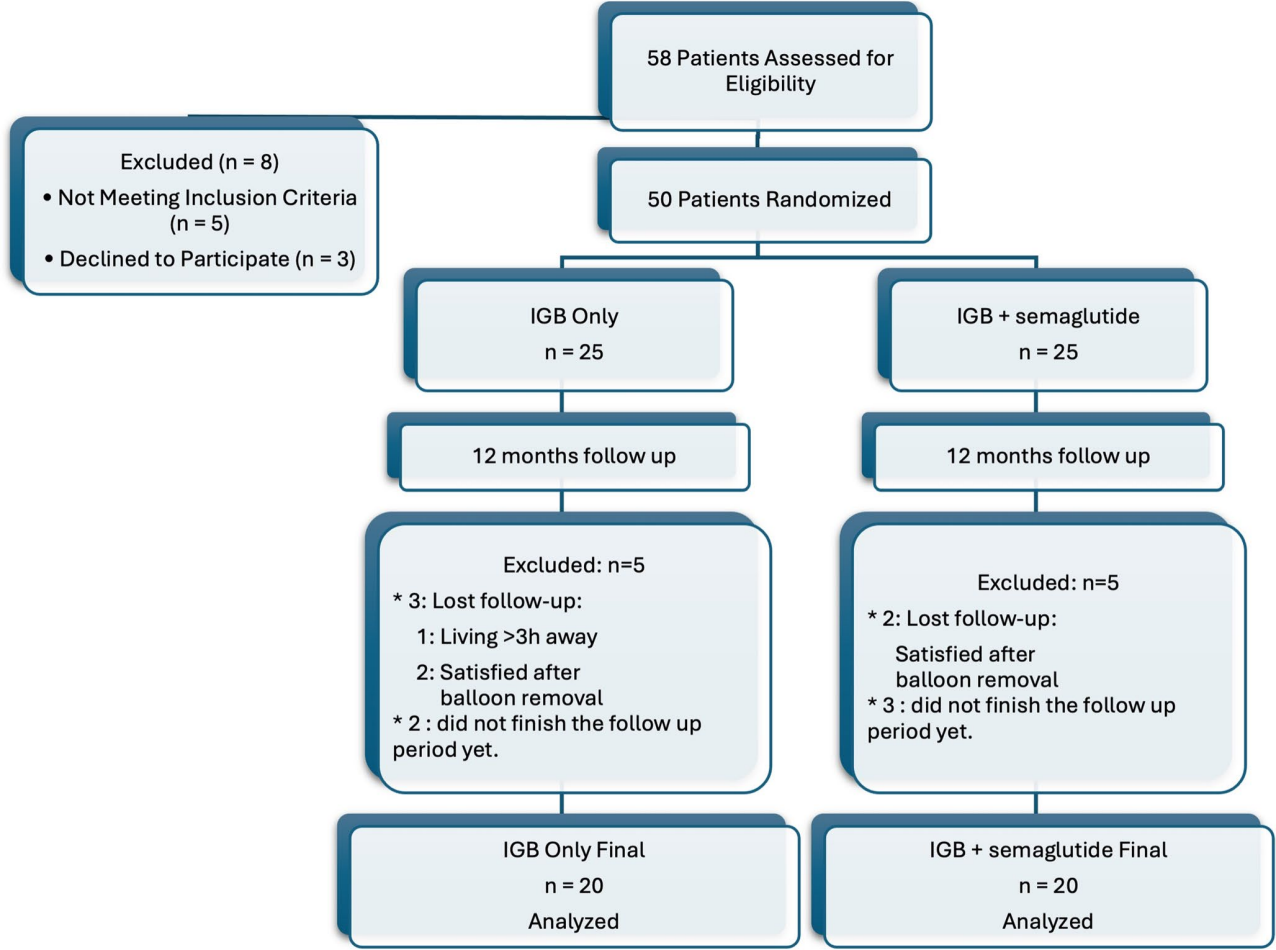
CONSORT flow diagram

A total of 40 patients were ultimately enrolled in the study and evenly assigned to two groups: IGB only and IGB + semaglutide, with 20 patients in each group. Initially, approximately 25 patients were recruited per group to account for potential withdrawals or loss to follow-up. During the study, 3 patients from the IGB-only group and 2 patients from the IGB + semaglutide group discontinued follow-up after balloon removal. One patient cited living more than three hours from the center as the reason, while the other four voluntarily withdrew because they were satisfied with their weight loss outcomes and felt no further medical supervision was necessary. Importantly, these voluntary discontinuations were based on perceived satisfactory results rather than inadequate weight loss, minimizing potential bias. The study concluded once 12-month follow-up data were obtained for 20 patients in each group. (Fig. 1)

### Demographic and Baseline Anthropometric

There were no statistically significant differences between the groups in baseline characteristics, including gender distribution, age, smoking status, diabetes prevalence, baseline anthropometrics, and ideal body weight. Gender distribution in the IGB-only group was 35.0% male and 65.0% female, while the IGB + semaglutide group comprised 30.0% male and 70.0% female (*p* = 0.736). The mean age was similar between groups (36.90 ± 9.16 years in the IGB only group vs. 36.20 ± 8.43 years in the IGB + semaglutide group, *p* = 0.803). No significant differences were noted in smoking status (5.0% vs. 10.0%, *p* = 1.000) or diabetes prevalence (30.0% vs. 20.0%, *p* = 0.465). At baseline, there were no statistically significant differences between the two groups regarding weight, height, or BMI. The mean baseline weight was 115.8 ± 9.94 kg in the IGB only group and 117.1 ± 9.73 kg in the IGB + semaglutide group (*p* = 0.669). Mean height and BMI were also comparable (*p* = 0.884 and *p* = 0.257, respectively). Regarding ideal body weight, both groups were comparable with mean values of 74.82 ± 7.57 kg in the IGB only group and 74.45 ± 6.30 kg in the IGB + semaglutide group (*p* = 0.866). (Table 1).

**Table 1:** Comparison between the two studied groups according to demographic and baseline anthropometric characteristics

		IGB-only (*n* = 20)	IGB + semaglutide (*n* = 20)	Test of Sig.	*p*	Effect size	Difference (95% C.I)
	Gender						
	Male	7 (35.0%)	6 (30.0%)	χ^2^=0.114	0.736	𝜑=0.053	0.05 (0.007–0.09)
	Female	13 (65.0%)	14 (70.0%)				
	Age						
	Min. – Max.	20.0–55.0	24.0–51.0	t = 0.251	0.803	d = 0.080	0.70 (−4.94–6.34)
	Mean ± SD.	36.90 ± 9.16	36.20 ± 8.43				
	Median (IQR)	37.50 (31.0–41.0)	35.0 (29.50–42.0)				
	Smoking	1 (5.0%)	2 (10.0%)	χ^2^ = 0.360	^FE^*p*=1.000	𝜑=0.095	0.05 (−0.04 − 0.06)
	Diabetes	6 (30.0%)	4 (20.0%)	χ^2^ = 0.533	0.465	𝜑=0.115	0.10 (0.06–0.14)
	Ideal weight						
	Min. – Max.	63.84–90.92	64.64–89.78	t = 0.170	0.866	d = 0.053	0.38 (−4.08–4.83)
	Mean ± SD.	74.82 ± 7.57	74.45 ± 6.30				
	Median (IQR)	73.02 (69.97–81.0)	73.36(70.48–76.17)				
Before	Weight						
	Min. – Max.	98.23–139.4	101.3–136.8	t = 0.430	0.669	d = 0.132	1.34 (4.96–7.64
	Mean ± SD.	115.8 ± 9.94	117.1 ± 9.73				
	Median (IQR)	113.7 (110.7–123.0)	116.7 (108.9–123.1)				
	Height						
	Min. – Max.	159.8–190.7	160.8–189.5	t = 0.147	0.884	d = 0.050	0.37 (−4.73–5.47)
	Mean ± SD.	172.8 ± 8.67	172.4 ± 7.20				
	Median (IQR)	170.9(167.3–180.0)	171.3(167.9–174.6)				
	BMI						
	Min. – Max.	35.62–41.91	36.86–42.82	t = 1.151	0.257	d = 0.363	0.61 (0.46–1.69)
	Mean ± SD.	38.75 ± 1.45	39.36 ± 1.88				
	Median (IQR)	38.55 (38.22–39.67)	39.37 (37.81–41.30)				

*IQR* Interquartile range, *SD* Standard deviation, *t* Student t-test

*χ* ^2^ Chi-square test, *FET* Fisher Exact test, *BMI* Body Mass Index

*d* Cohen’s d, 𝜑 Phi

*P* p-value for comparing the two studied groups

These findings demonstrate that the two groups were well matched at baseline, with no statistically significant differences observed. The similarity in these key demographic and clinical characteristics indicates that randomization was effective. Additionally, the lack of significant differences in baseline weight, height, BMI, and ideal body weight further supports the comparability of the groups. This balanced distribution of baseline characteristics minimizes potential confounding factors and strengthens the validity of attributing any subsequent differences in outcomes to the interventions under investigation rather than to pre-existing differences.

### Complications

During the study, no serious complications occurred in either group that required discontinuation of semaglutide or removal of the gastric balloon. The most frequently reported adverse events were nausea and vomiting, affecting 7 patients (35.0%) in the IGB-only group and 10 patients (50.0%) in the IGB + semaglutide group. Although these events were more common in the combination group, the difference between groups was not statistically significant (χ² = 0.921, *p* = 0.337). Symptoms were generally mild and effectively managed with supportive care, including the use of antiemetics administered according to clinical need. All patients also received standard post-IGB insertion medications, such as proton pump inhibitors and analgesics, to enhance tolerance and reduce early side effects. Close monitoring allowed prompt identification and management of gastroparesis-related symptoms, ensuring patient safety while maintaining adherence to the prescribed treatment regimen. Overall, both the gastric balloon and semaglutide injections were well tolerated, and adherence remained high in both groups. (Table 2).

**Table 2:** Comparison between the two groups studied according to the complications

	IGB-only (*n* = 20)	IGB + semaglutide (*n* = 20)	Test of Sig.	*p*	Effect size	Difference (95% C.I)
Serious complications	0 (0.0%)	0 (0.0%)	–	–	–	
Nausea & vomiting	7 (35.0%)	10 (50.0%)	χ^2^ = 0.921	0.337	𝜑=0.152	0.15 (0.10–0.20)

*χ* ^2^ Chi-square test, *P* p-value for comparing the two studied groups

𝜑 Phi

### Anthropometric Changes

Anthropometric parameters were assessed at baseline and at 3, 6, and 12 months following intervention for both groups. At baseline, in the IGB-only group, the mean weight was 115.8 ± 9.94 kg, while the IGB + semaglutide group had a mean weight of 117.1 ± 9.73 kg. The mean height was 172.8 ± 8.67 cm in the IGB only group and 172.4 ± 7.20 cm in the IGB + semaglutide group. For BMI, the IGB only group demonstrated a mean of 38.75 ± 1.45, compared to 39.36 ± 1.88 in the IGB + semaglutide group. (Table 1)

After 3 months, both groups showed reductions in weight and BMI, but the differences between them were not statistically significant. Mean weight was 104.9 ± 9.54 kg in the IGB only group and 103.7 ± 9.99 kg in the IGB + semaglutide group (*p* = 0.699), while BMI was 35.13 ± 1.67 and 34.85 ± 1.97, respectively (*p* = 0.626). By 6 months, statistically significant differences emerged. The IGB + semaglutide group showed a greater reduction in both weight and BMI compared to the IGB only group. Mean weight decreased to 97.41 ± 9.38 kg in the IGB only group and to 88.00 ± 8.64 kg in the IGB + semaglutide group (*p* = 0.002). Similarly, mean BMI dropped to 32.59 ± 1.49 in the IGB only group and to 29.54 ± 1.23 in the IGB + semaglutide group (*p* < 0.001). At 12 months, the differences were even more pronounced. The IGB + semaglutide group maintained significantly greater weight loss, with a mean weight of 84.07 ± 7.58 kg compared to 100.2 ± 9.27 kg in the IGB only group (*p* < 0.001). Correspondingly, BMI was significantly lower in the IGB + semaglutide group (28.23 ± 1.08) than in the IGB only group (33.53 ± 1.26) (*p* < 0.001). (Table 3)

**Table 3:** Comparison between the two groups studied according to anthropometric measurements at baseline, 3, 6, and 12 months

		IGB-only (n = 20)	IGB + semaglutide (n = 20)	Test of Sig.	p	Effect size	Difference (95% C.I)
Before	Weight						
	Min. – Max.	98.23–139.4	101.3–136.8	t = 0.430	0.669	d = 0.132	1.34 (4.96–7.64
	Mean ± SD.	115.8 ± 9.94	117.1 ± 9.73				
	Median (IQR)	113.7 (110.7–123.0)	116.7 (108.9–123.1)				
	BMI						
	Min. – Max.	35.62–41.91	36.86–42.82	t = 1.151	0.257	d = 0.363	0.61 (0.46–1.69)
	Mean ± SD.	38.75 ± 1.45	39.36 ± 1.88				
	Median (IQR)	38.55 (38.22–39.67)	39.37 (37.81–41.30)				
After 3 months	Weight						
	Min. – Max.	90.73–128.7	88.43–125.4	t = 0.390	0.699	d = 0.123	1.20 (−5.05–7.46)
	Mean ± SD.	104.9 ± 9.54	103.7 ± 9.99				
	Median (IQR)	103.3 (97.89–110.4)	101.1 (97.32–109.7)				
	BMI						
	Min. – Max.	31.40–38.30	32.33–38.85	t = 0.492	0.626	d = 0.153	0.28 (−0.88–1.45)
	Mean ± SD.	35.13 ± 1.67	34.85 ± 1.97				
	Median (IQR)	35.17 (34.48–36.35)	34.36 (33.04–36.03)				
After 6 months	Weight						
	Min. – Max.	81.33–119.8	76.25–106.9	t = 3.297^*^	0.002^*^	d = 1.044	9.40 (3.63–15.18)
	Mean ± SD.	97.41 ± 9.38	88.00 ± 8.64				
	Median (IQR)	95.64 (90.91–105.5)	87.38 (82.24–93.60)				
	BMI						
	Min. – Max.	29.47–35.19	27.65–31.62	t = 7.066^*^	< 0.001^*^	d = 2.232	3.05 (2.18–3.93)
	Mean ± SD.	32.59 ± 1.49	29.54 ± 1.23				
	Median (IQR)	32.80 (31.87–33.60)	29.43 (28.68–30.55)				
After 12 months	Weight						
	Min. – Max.	83.24–120.3	72.97–98.74	t = 6.022	< 0.001^*^	d = 1.905	16.13 (10.71–21.55)
	Mean ± SD.	100.2 ± 9.27	84.07 ± 7.58				
	Median (IQR)	97.73 (95.71–109.13)	82.44 (79.24–89.28)				
	BMI						
	Min. – Max.	30.37–35.38	26.61–30.26	t = 14.259	< 0.001^*^	d = 4.517	5.29 (4.54–6.04)
	Mean ± SD.	33.53 ± 1.26	28.23 ± 1.08				
	Median (IQR)	33.48 (32.96–34.39)	28.10 (27.44–28.97)				

*IQR* Interquartile range, *SD* Standard deviation, d Cohen’s d

*t* Student t-test, *BMI* Body Mass Index

*P* p-value for comparing the two studied groups, * Statistically significant at p ≤ 0.05

These results indicate that while both treatment modalities led to significant reductions in weight and BMI over time, the addition of semaglutide to IGB therapy yielded significantly superior outcomes, especially evident at 6 and 12 months of follow-up.

### Weight Loss Analysis

Significant differences were observed between the two groups in terms of weight loss at both 6 and 12 months, as well as in weight regain after intragastric balloon (IGB) removal. At 6 months, the IGB + semaglutide group achieved a markedly greater mean weight loss compared to the IGB only group, with values of 29.09 ± 3.45 kg and 18.35 ± 2.80 kg, respectively (*p* < 0.001). After 12 months, the difference widened further. The IGB + semaglutide group maintained a significantly higher weight loss with a mean of 33.03 ± 3.55 kg, while the IGB only group showed a reduced mean loss of 15.56 ± 2.50 kg (*p* < 0.001). The Total Body Weight Loss percentage (TBWL%) was significantly higher in the IGB + semaglutide group compared to the IGB only group (28.21 ± 2.11% vs. 13.47 ± 2.06%, *p* < 0.001). Similarly, the Excess Weight Loss percentage (EWL%) was markedly greater in the IGB + semaglutide group (77.85 ± 5.55%) than in the IGB only group (38.14 ± 5.82%, *p* < 0.001). (Table 4)

**Table 4:** Comparison between the two groups studied according to changes in weight

	IGB-only (*n* = 20)	IGB + semaglutide (*n* = 20)	Test of Sig	*p*	Effect size	Difference (95% C.I)
Weight loss after 6 months						
Min. – Max.	13.21–23.32	20.59–33.66	t = 10.813	< 0.001^*^	d = 3.418	10.74 (8.73–12.75)
Mean ± SD.	18.35 ± 2.80	29.09 ± 3.45				
Median (IQR)	17.79(16.43–20.82)	28.95(26.87–32.14)				
Weight loss after 12 months						
Min. – Max.	11.05–19.41	26.90–38.35	t = 17.998	< 0.001^*^	d = 1070.751	17.47 (15.50–19.43)
Mean ± SD.	15.56 ± 2.50	33.03 ± 3.55				
Median (IQR)	15.24(13.87–17.88)	32.80(30.38–36.39)				
Weight regains after removal						
Min. – Max.	0.26–6.42	−8.22 – −1.07	U = 0.000	< 0.001^*^	η2 = 0.750	6.690 (5.270–8.010)
Mean ± SD.	2.79 ± 1.74	−3.94 ± 2.16				
Median (IQR)	2.21 (1.84–3.84)	−3.70 (−5.15 – −2.25)				
12-Month TBWL %						
Min. – Max.	10.46–17.37	23.77–32.10	t = 22.369	< 0.001^*^	d = 7.069	14.75 (−13.41– 16.08)
Mean ± SD.	13.47 ± 2.06	28.21 ± 2.11				
Median (IQR)	13.40(11.36–15.01)	27.97(27.21–29.89)				
12-Month EWL %						
Min. – Max.	29.86–49.48	68.72–87.26	t = 22.099	< 0.001^*^	d = 6.983	39.71 (36.08–43.35)
Mean ± SD.	38.14 ± 5.82	77.85 ± 5.55				
Median (IQR)	37.62(33.11–43.83)	78.53(72.96–81.34)				

*IQR* Interquartile range, *SD* Standard deviation

*t* Student t-test *U* Mann-Whitney test

*d* Cohen’s d *η2* eta square

*TBWL%* Total Body Weight Loss percentage, *EWL%* Excess Weight Loss percentage

*P* p-value for comparing between the two studied groups, * Statistically significant at *p* ≤ 0.05

Regarding weight regain after IGB removal, the IGB-only group exhibited a *positive* regain, with a mean increase of 2.79 ± 1.74 kg, indicating partial reversal of weight loss. In contrast, the IGB + semaglutide group continued to experience weight reduction even after balloon removal, with a mean change of −3.94 ± 2.16 kg (*p* < 0.001). This analysis highlights the significant and sustained benefit of continued semaglutide therapy. (Table 4).

These results confirm that combining semaglutide with IGB therapy not only leads to greater initial weight loss but also prevents post-balloon weight regain.

## Discussion

Given the significant increase in prevalence and burden of obesity globally, there is a need to explore and establish available treatment modalities and their outcomes. As of 2021, approximately 1 in 8 people worldwide were living with obesity [11]. With 42% of United States adults classified as obese and obesity-related healthcare expenses estimated at $173 billion annually, effective and sustainable interventions are necessary [5]. Currently, most studies assess the effectiveness of individual therapies, leaving limited evidence on the results of combination therapy. This study was conducted to evaluate the efficacy of combining endoscopic and pharmaceutical interventions, including intragastric balloon and semaglutide injections. Our results demonstrate the following findings: Both the IGB only group and the IGB + semaglutide group significantly lost weight compared to baseline; the IGB + semaglutide group had a significantly higher total body weight loss (TBWL) and excess weight loss (EWL) percentage when compared to the IGB only group, primarily at 6 and 12 months; the IGB only group had significant weight regain in comparison to the IGB + semaglutide group after intragastric balloon removal.

The IGB only group and the IGB + semaglutide group demonstrated statistically significant reductions compared to baseline body weight of 9.41% and 11.44% at 3 months, 15.88% and 24.85% at 6 months, and 13.47% and 28.21% at 12 months, respectively. In comparison, a multicenter clinical trial on IGB use reported a total body weight loss of 8.5% at 3 months, 11.8% at 6 months, and 13.3% at 12 months, while semaglutide studies have shown approximately 10.9% weight loss at 6 months and 13.9% at 12 months [12, 13]. Both the IGB only group and the IGB + semaglutide group achieved the most substantial weight reduction during the first six months, aligning with the established therapeutic window for IGB therapy [14]. The IGB only group achieved its lowest weight at 6 months. The IGB + semaglutide group achieved maximal weight loss at 12 months following balloon removal. This pattern is likely due to the complementary mechanisms of both therapies. The IGB leads to weight reduction by mechanically obstructing gastric volume and stimulating satiety via mechanoreceptors [15]. Following balloon removal, semaglutide sustains satiety and appetite suppression via GLP-1 receptor activation [16]. The IGB + semaglutide group continued weight loss demonstrates the efficacy of semaglutide therapy, and its ability to not only sustain but also further weight loss subsequent to balloon removal.

Semaglutide injections were given at 2, 3, and 4 months, with injections at the 5th and 6th month withheld, followed by a booster dose at the 7th and 8th month. This sustained weight loss suggests semaglutide therapy may overcome the 6-month plateau following 10–13% weight reduction often observed in IGB ballooning due to metabolic adaptation and hormonal changes [5, 17]. This synergistic effect persisted following balloon removal and despite semaglutide dosage reduction, emphasizing the efficacy and cost efficiency of this combined therapy. These results demonstrate the advantages of combined IGB and semaglutide therapy on early weight loss and sustained appetite suppression compared to IGB alone.

**Fig. 2:**
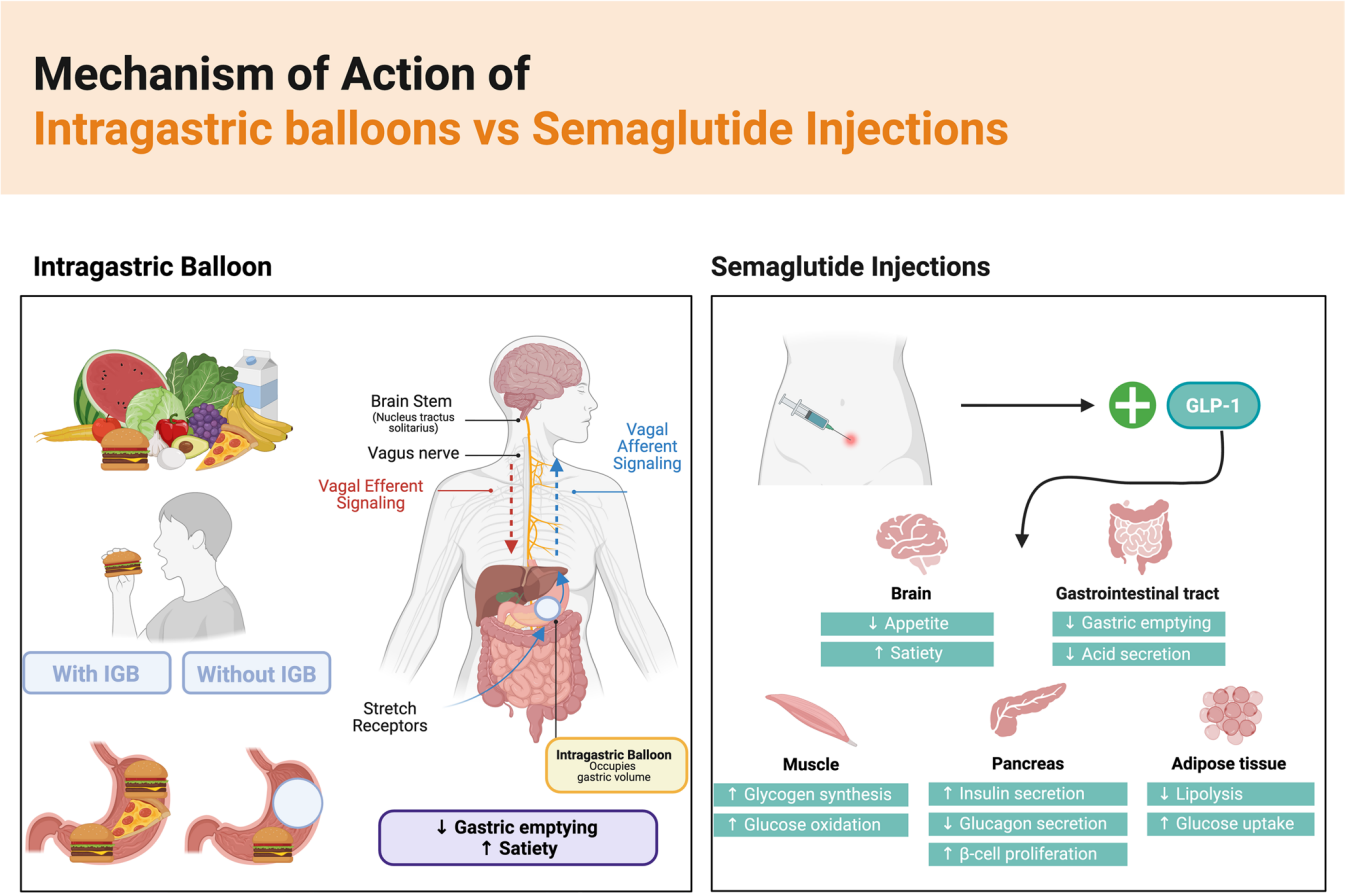
Mechanism of action for Intragastric Balloon (IGB) therapy versus Semaglutide injections. The left panel illustrates the decreased gastric volume due to IGB, which leads to reduced food intake. It also demonstrates the effect IGB has on stretch receptors in the stomach, activating afferent vagal signaling to alert the nucleus tractus solitarius in the brain stem, which subsequently triggers vagal efferent firing, decreasing gastric emptying and increasing satiety. The right panel demonstrates the effects of semaglutide injections as a GLP-1 agonist, which enhances satiety and reduces appetite via central effects, delays gastric emptying, and decreases gastric acid secretion. Peripheral effects include increased glycogen synthesis and glucose oxidation in muscle, enhanced insulin secretion and β-cell proliferation with reduced glucagon secretion in the pancreas, and decreased lipolysis with increased glucose uptake in adipose tissue. Both interventions reduce energy intake through complementary mechanisms. Created in BioRender. Basson, M. (2025) https://BioRender.com/szuyr7g

When comparing both treatment groups, the IGB + semaglutide group had a significantly higher TBWL and EWL than the IGB only group, specifically at 6 and 12 months. Even though both groups did lose significant weight at 3 months compared to baseline, the IGB + semaglutide group showed an increased weight loss that was not statistically significant in comparison to the IGB only group. Semaglutide induces weight loss through its interactions with GLP-1 receptors that directly and indirectly regulate the hypothalamus and hindbrain, which are brain areas involved in appetite mediation [18]. Semaglutide’s effects on weight reduction are commonly observed within the first four weeks of treatment, yet its maximal effect is achieved by week 60 [17, 19]. This may explain why weight reduction at 3 months was evident in both the IGB-only group and the IGB + semaglutide group, yet this difference was not found to be significant until 6 and 12 months of therapy. Placement of the intragastric balloon triggers weight loss through a restrictive mechanism using space-occupancy [20]. This modifies gastric accommodation and emptying, which further prompts feelings of satiety through gastrointestinal neurohormonal pathways [20]. Given semaglutide’s ability to delay gastric emptying and suppress appetite [5], along with the intragastric balloon’s space-occupying mechanism, this treatment approach may exhibit a synergistic effect on weight loss. (Fig. 2) This synergistic effect likely triggers greater weight reduction than the use of either semaglutide or IGB alone, which is evident with the significantly higher TBWL and EWL evident in the IGB + semaglutide group.

A notable outcome in this study was the difference in weight loss trajectory following intragastric balloon removal at 6 months. The IGB-only group regained 15.2% of previously lost weight while the IGB + semaglutide group lost an additional 13.5% of previously lost weight. On comparing the IGB + semaglutide group and the IGB only group from a standpoint of weight gain, the IGB only group showed a statistically significant weight regain compared to the IGB + semaglutide group. These findings align with reported weight regain in 76% of patients after balloon removal, highlighting the need for adjunctive weight loss intervention for sustained outcomes [21]. Weight regain following balloon removal may be attributed to interindividual variation in metabolic efficiency, particularly resting metabolic rate, of which 40% is attributed to genetics [22]. Given these factors, patients may require longer to achieve sustainable metabolic adjustments. Semaglutide therapy offers a complementary yet distinct approach to weight loss compared to the IGB balloon, as it reduces energy intake at the hormonal level via the brain’s reward and motivation pathway rather than mechanically [23, 24]. Semaglutide therapy also does not alter resting metabolic rate, therefore justifying subsequent therapy to adjust energy intake during metabolic rate adjustments to support long-term weight loss [23, 24]. 

Nausea and vomiting were common adverse effects reported by both the IGB only group and the IGB + semaglutide group. Although not significant in comparison to the IGB only group, a greater incidence was reported by patients in the IGB + semaglutide group. The nausea and vomiting reported by patients in the IGB only group may be explained by IGB’s effect on gastric wall tension, which is influenced by factors of gastric wall stretch and tone [25]. Activating gastric mechanoreceptors that respond to changes in stretch regulates the intensity of sensation felt after gastric filling [25]. While eating, an accommodation reflex allows various volumes to enter the stomach without a drastic rise in pressure [25]. This is achieved with the gradual relaxation of the stomach walls [25]. However, due to the space-occupying nature of IGB, the stomach walls are unable to fully relax, which may be the mechanism by which IGB patients report symptoms of nausea and vomiting. On the other hand, semaglutide activates central GLP-receptors located in the area postrema and nucleus tractus solitarius, which are both brain regions involved in the emetic response [26]. Additionally, semaglutide delays gastric emptying, which in turn increases gastric distention and vagal afferent stimulation, causing symptoms of nausea [26]. Given semaglutide’s contribution to nausea and vomiting, its combination with IGB may amplify emetic symptoms and therefore explain the higher occurrence observed in the IGB + semaglutide group patients.

Several limitations in our research should be discussed and taken into account when guiding future research. The sample size was small, with 20 patients per treatment group and a total of 40 patients. Due to the small size of this cohort, this may limit the statistical power to detect sudden differences. However, baseline demographics, including gender, age, smoking, diabetes, and ideal weight, were not found to be significant between the groups. Additionally, this study was limited by a follow-up duration of only 12 months and was conducted at a single center, rather than multiple centers. This may influence the generalizability of the study. Another limitation in the study was that there was no group with patients just on semaglutide or a placebo. This would have allowed for a comparison between each intervention individually and then a combination of both treatments. Additionally, there was a limitation related to diet, as participants of the study were Egyptian and may have genetic profiles and diets that differ from other populations. These factors may influence outcomes if this study were to be replicated. However, the diet that participants were placed on was a standardized and structured plan that can be broadly applied to other populations, therefore reducing variability. Furthermore, other than weight loss, weight regain, and complications, there were no other metrics assessed, such as waist circumference.

An additional limitation was the lack of a follow-up after the 12 months period, as additional variables may have varied past this point.

## Conclusion

This study demonstrates that adjunctive semaglutide therapy with intragastric balloon (IGB) enhances both the magnitude of short-term weight loss, while enhancing the sustainability achieved with intragastric ballooning alone. While IGB has demonstrated efficacy for short-term weight loss, the addition of semaglutide injections extends weight loss beyond IGB’s typical therapeutic window, potentially mitigating the 6-month weight plateau observed with IGB therapy alone. Combining the two therapies optimizes mechanical and hormonal mechanisms to reduce energy intake, providing a synergistic effect to enhance short-term weight loss at 6 and 12 months while counteracting weight regain at 6 months. These results support the addition of semaglutide injections following intragastric ballooning to improve patient outcomes. This therapeutic model provides a promising, evidence-based solution to this growing epidemic by combining currently available and approved weight loss methods to optimize patient results and long-term outcomes. Future research is warranted to examine more long-term outcomes of this synergistic therapy at 2, 5, and 10 years to assess the therapeutic efficacy across various populations.

## Data Availability

The data are available upon reasonable request from the corresponding author.
